# Heart rate and cardiac autonomic responses to concomitant deep breathing, hand grip exercise, and circulatory occlusion in healthy young adult men and women

**DOI:** 10.1186/s40659-021-00355-1

**Published:** 2021-09-26

**Authors:** David C. Andrade, Claudia Melipillan, Camilo Toledo, Angélica Rios-Gallardo, Noah J. Marcus, Fernando C. Ortiz, Gonzalo Martinez, Paula Muñoz Venturelli, Rodrigo Del Rio

**Affiliations:** 1 Laboratory of Cardiorespiratory Control, Department of Physiology, Pontificia Universidad Católica de Chile, Santiago, Chile; 2 Centro de Fisiología Y Medicina de Altura, Departamento Biomedico, Facultad de Ciencias de La Salud, Universidad de Antofagasta, Antofagasta, Chile; 3 Corporación de Rehabilitación Club de Leones Cruz del Sur, Punta Arenas, Chile; 4 Centro de Excelencia en Biomedicina de Magallanes (CEBIMA), Universidad de Magallanes, Punta Arenas, Chile; 5 Dept. of Physiology and Pharmacology, Des Moines University, Des Moines, IA USA; 6 Mechanism of Myelin Formation and Repair Laboratory, Instituto de Ciencias Biomédicas, Facultad de Ciencias de Salud, Universidad Autónoma de Chile, Santiago, Chile; 7 Division of Cardiovascular Diseases, Pontificia Universidad Católica de Chile, Santiago, Chile; 8 Centro de Estudios Clínicos, Instituto de Ciencias E Innovación en Medicina, Facultad de Medicina Clínica Alemana, Universidad del Desarrollo, Santiago, Chile; 9 Centro de Envejecimiento Y Regeneración (CARE), Pontificia Universidad Católica de Chile, Santiago, Chile

**Keywords:** Deep breathing, Isometric handgrip, Cardiac autonomic control, Sympathetic, Parasympathetic

## Abstract

**Background:**

Deep breathing (DB) and handgrip (HG) exercise -with and without circulatory occlusion (OC) in muscle-, have been shown to have beneficial effects on cardiovascular function; however, the combination of these maneuvers on heart rate (HR) and cardiac sympathovagal balance have not been previously investigated. Therefore, the aim of the present study was to evaluate the effect of simultaneous DB, HG, and OC maneuvers on the sympathovagal balance in healthy women and men subjects.

**Methods and results:**

Electrocardiogram and ventilation were measured in 20 healthy subjects (Women: n = 10; age = 27 ± 4 years; weight = 67.1 ± 8.4 kg; and height = 1.6 ± 0.1 m. Men: n = 10; age = 27 ± 3 years; weight = 77.5 ± 10.1 kg; and height = 1.7 ± 0.1 m) at baseline and during DB, DB + HG, or DB + HG + OC protocols. Heart rate (HR) and respiratory rate were continuously recorded, and spectral analysis of heart rate variability (HRV) were calculated to indirectly estimate cardiac autonomic function. Men and women showed similar HR responses to DB, DB + HG and DB + HG + OC. Men exhibited a significant HR decrease following DB + HG + OC protocol which was accompanied by an improvement in cardiac autonomic control evidenced by spectral changes in HRV towards parasympathetic predominance (HRV High frequency: 83.95 ± 1.45 vs. 81.87 ± 1.50 n.u., DB + HG + OC vs. baseline; p < 0.05). In women, there was a marked decrease in HR after completion of both DB + HG and DB + HG + OC tests which was accompanied by a significant increase in cardiac vagal tone (HRV High frequency: 85.29 ± 1.19 vs. 77.93 ± 0.92 n.u., DB + HG vs. baseline; p < 0.05). No adverse effects or discomfort were reported by men or women during experimental procedures. Independent of sex, combination of DB, HG, and OC was tolerable and resulted in decreases in resting HR and elevations in cardiac parasympathetic tone.

**Conclusions:**

These data indicate that combined DB, HG and OC are effective in altering cardiac sympathovagal balance and reducing resting HR in healthy men and women.

## Background

Sympathovagal imbalance is a common pathophysiological feature of many cardiovascular diseases, and thus an important target of therapeutic strategies [1]. Standard-of-care treatments for cardiovascular diseases have varying degrees of efficacy in attenuating sympathovagal imbalance and therefore additional pharmacological and/or non-pharmacological therapeutic options are desirable. Therefore, considering that autonomic imbalance is related to mortality and severity in different pathological conditions [2–4], studies looking at non-pharmacological and cost-effective strategies intended to improve cardiac autonomic fitness are needed.

Voluntary slow breathing exercises have been proposed as a potential non-pharmacological approach to improving outcomes in cardiovascular disease due to their effect on sympathovagal tone [5]. Voluntary slow breathing is generally characterized by decreased respiratory rate with increased respiratory amplitude. Previous studies have shown that a respiratory rate of ~ 0.1 Hz produces a strong entrainment of autonomic cardiovascular and respiratory rhythms as determined by spectral analysis of heart rate variability (HRV) [6]. The rhythmic entrainment and related salutary effects of deep breathing (DB) are likely mediated by activation of the Hering-Breuer reflex, related improvements in baroreflex sensitivity, and increased cardiac vagal tone and decreased cardiac sympathetic tone [6, 7]. While there is evidence that DB offers a non-pharmacological strategy to improve cardiac autonomic function [8], there are other non-pharmacological therapies that have been shown to have similar effects, such as isometric handgrip exercise [9].

Isometric exercise involves sustained contraction against an immovable load or resistance with no or minimal change in length of the involved muscle group. Importantly, it has been shown that this training modality produces significant and clinically meaningful reductions in heart rate (HR) and blood pressure (BP) [10], and better cardiovascular outcomes compared to dynamic aerobic and/or resistance exercise [10]. The beneficial effects of HG exercise on cardiovascular function are likely related to the activation of the mechano-metaboreflex during muscle contraction [8, 11, 12]. Mechanical compression and metabolite accumulation within the muscle tissue during HG stimulates mechano/metabo sensitive afferent fibers (groups III and IV) which promote sympathoexcitation and parasympathetic withdrawal [8, 11, 12]. Interestingly, it has been proposed that circulatory occlusion (OC) through external compression of muscle would likely elicit comparable effects on the neural regulation of cardiovascular function as the ones observed during HG exercise [12].

Despite the fact that several studies indicate DB, HG, and OC individually may exert similar salutary effects on autonomic control of cardiovascular function in physiological and pathophysiological states, there are currently no studies that have addressed the potential additive or synergistic effect of these approaches in combination. Therefore, the aim of the present study was to identify suitability and feasibility of the combination of DB, HG and OC as an affordable strategy to improve HR control through cardiac sympathovagal modulation. Gender differences on the effects of the combined maneuvers on cardiac sympathovagal balance were also determined.

## Results

Body mass and body height were significantly different between men and women (p < 0.01); however, body mass index (BMI) was similar between groups (Table 1). Similarly, age, systolic blood pressure (SBP), diastolic BP (DBP), pulse pressure (PP), and mean arterial BP (MABP) were not different between groups (Table 1). In terms of physical activity, 40% of the total participants (n = 8) were classified as sedentary and 60% (n = 12) declared to participate in recreational exercise activities 3–4 times per week (70 and 50% of men and women, respectively). Based on BMI, subjects were classified as follows: 13 normal range (6 men and 7 women), 5 overweight (3 men and 2 women), and 2 obese (1 man and 1 woman). No women were taking oral contraceptives during the study (Table 1).

**Table 1:** Demographic and Cardiovascular characteristics of study participants

	Men, n = 10	Women, n = 10
Age (years)	26.5 ± 3.5	27.5 ± 3.6
Body mass (kg)	77.5 ± 10.1	67.09 ± 8.4**
Body height (m)	1.76 ± 0.1	1.62 ± 0.1**
BMI (kg/m^2^)	24.9 ± 3.2	25.56 ± 3.8
SBP (mmHg)	120.6 ± 4.5	121.75 ± 9.7
DBP (mmHg)	76.83 ± 8.3	77.01 ± 11.7
PP (mmHg)	43.8 ± 6.5	44.8 ± 8.1
MABP (mmHg)	90.53 ± 6.5	90.99 ± 10.3
Healthy habits (n)		
Smoking	1	1
Comorbidities	0	0
Physical activity (n)		
Practice	7	5
Not Practice	3	5
Body Mass Index (n)		
Normal	6	7
Overweight	3	2
Obesity	1	1
Medication (n)		
Use	0	0
Not Use	10	10

Data are shown as mean ± standard deviation (SD) and as number of subjects (n)

*SBP* systolic blood pressure, *DBP* diastolic blood pressure, *PP* pulse pressure, *MABP* mean arterial blood pressure. Data analyzed by unpaired T-test

**p < 0.01

### Effects of DB, DB + HG and DB + HG + OC on HR

**Fig. 1:**
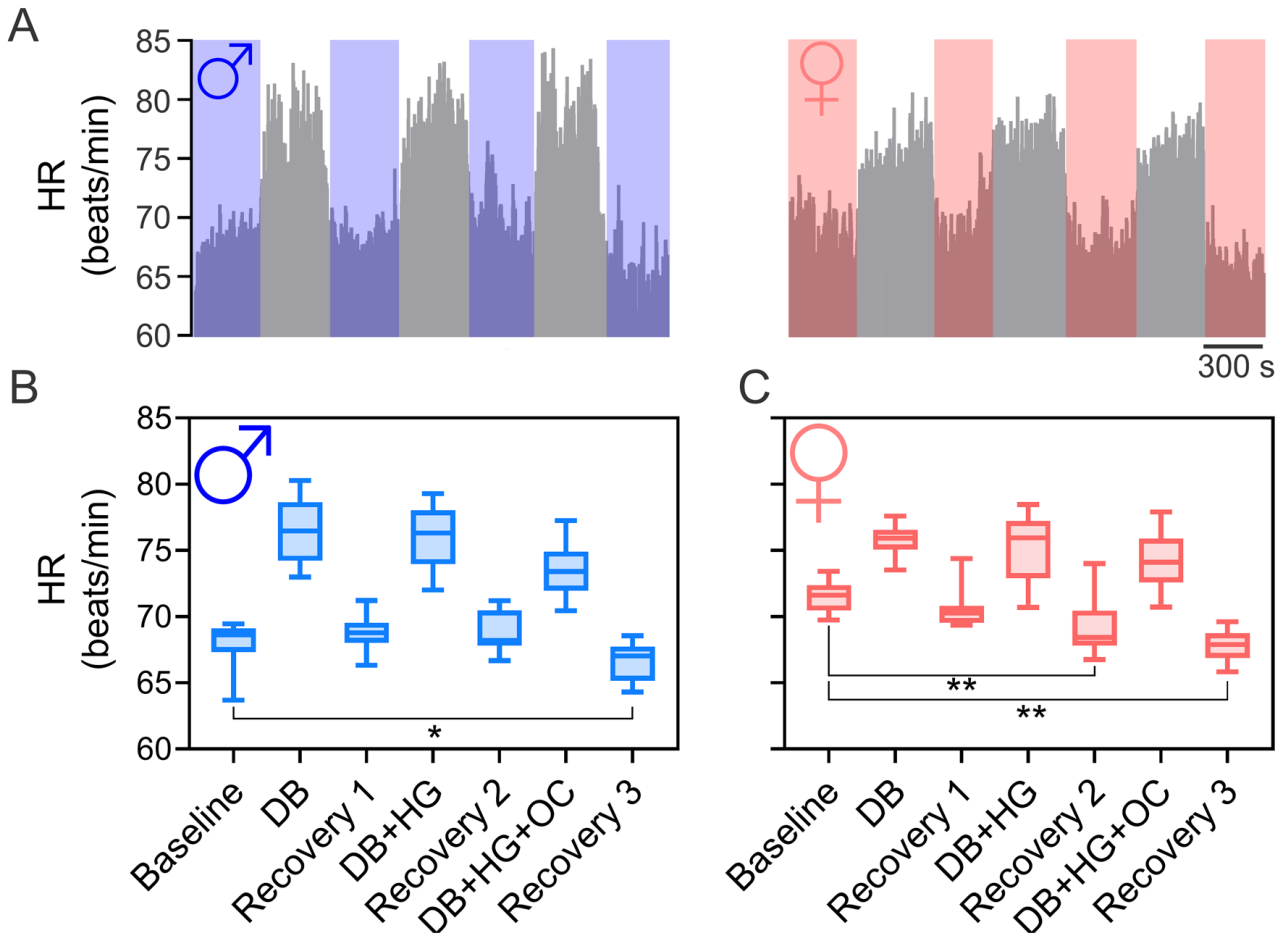
Effect of deep breathing (DB), DB + hand grip (DB + HG) and DB + HG + circulatory occlusion (DB + HG + OC) on heart rate response. **A** Representative HR response during DB, DB + HG, and DB + HG + OC from one man and one woman. Note that the decrease of HR during recovery period was more pronounced in women than men. **B** Summary results of HR response during the different interventions in men. Note that HR during the recovery period 3 (post DB + HG + OC) was significantly different compared to the Baseline period. **C** Summary results of HR response during the different maneuvers in women. Note that the HR during recovery period 2 (DB + HG) and 3 (post DB + HG + OC) were significantly different compared to the Baseline period. Data analyzed by one-way ANOVA, followed by Holm-Sidak post hoc. *, p < 0.05; **, p < 0.01

HR is showed in Fig. 1. No significant differences were observed between men and women at baseline (68.2 ± 3.9 vs. 71.5 ± 4.3 beats/min, respectively) (Fig. 1A–C). Compared to resting values, both men and women displayed slight differences in HR during the recovery phases following interventions. Men showed a significant decrease in HR during recovery 3 (post DB + HG + OC) compared to baseline values (66.6 ± 3.4 vs 68.2 ± 3.9 beats/min, Recovery 3 vs. Baseline, respectively, statistical power (SP): 0.78) (Fig. 1B). In addition, a decrease in HR was observed during recovery 2 (post DB + HG) and recovery 3 in women (post DB + HG + OC) (69.3 ± 2.2 vs. 67.8 ± 1.7 vs. 71.5 ± 4.3 beats/min, respectively, SP: 0.68) (Fig. 1C).

### Effects of DB, HG and OC on cardiac autonomic function

**Fig. 2:**
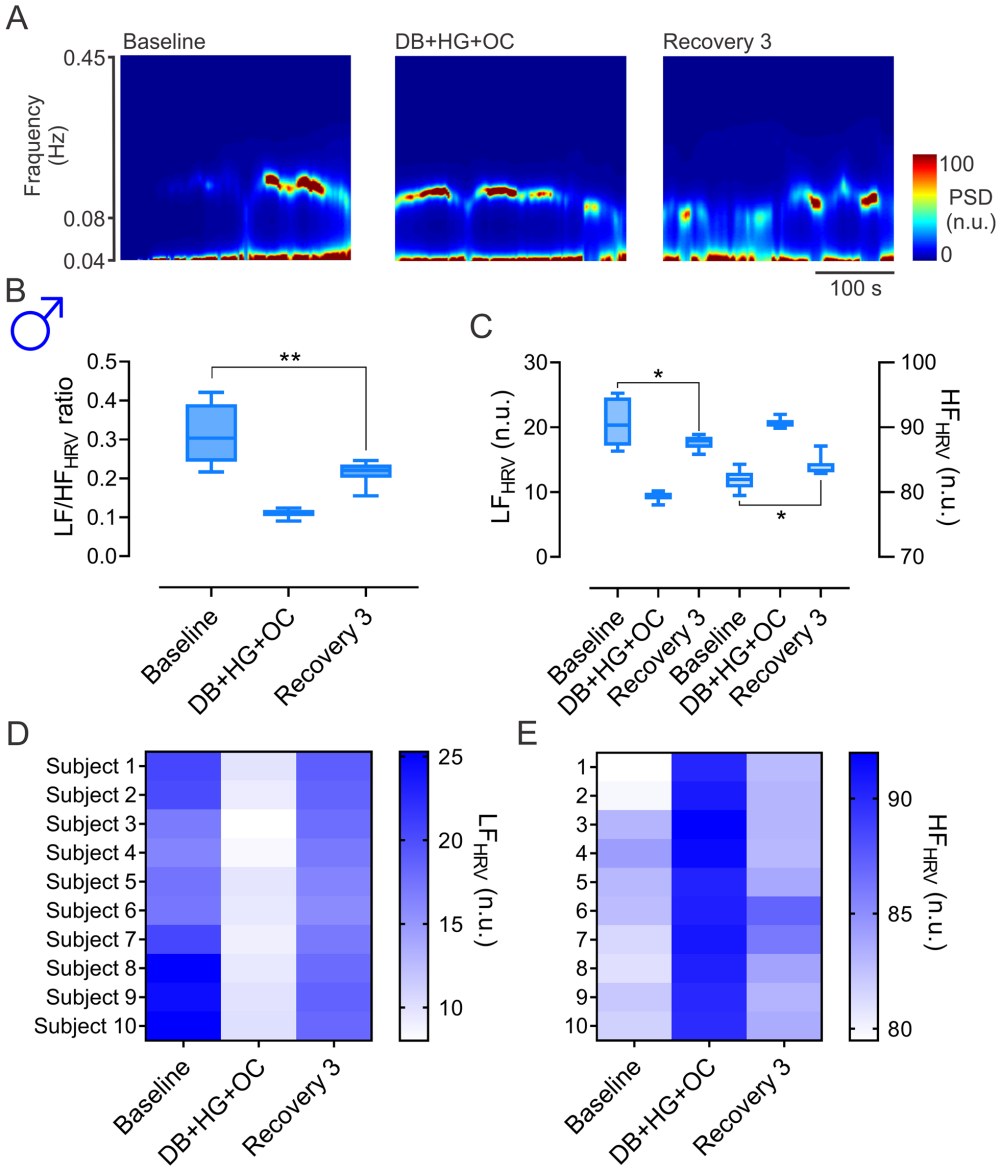
Effect of deep breathing (DB) + hand grip (DB + HG) + occlusion (DB + HG + OC) on heart rate variability (HRV) during recovery period 3 in men. **A** Representative time-varying spectrum of HRV during baseline period, DB + HG + OC and recovery period 3 from one man. Note that the power spectral density at low frequency (LF, 0.04–0.08 Hz) and high frequency (HF, 0.08–0.45 Hz) component of HRV, were decreased and increased, respectively, during recovery period 3 compared to the baseline. **B** Summary data of LF to HF ratio during baseline, DB + HG + OC, and during recovery period 3. Note that LF/HF ratio was significantly reduced in recovery period 3 compared to the baseline. **C** Summary data of LF and HF during baseline, DB + HG + OC, and during recovery period 3. **D**, **E** Interindividual response of LF and HF of HRV in men, respectively. Data analyzed by one-way ANOVA, followed by Holm-Sidak post hoc. *, p < 0.05; **, p < 0.01

We evaluated cardiac autonomic function through heart rate variability (HRV) analysis, during baseline and the recovery period after DB + HG + OC in men. We found that low frequency to high frequency (LF/HF) ratio was significantly decreased during recovery compared to baseline values (0.21 ± 0.02 vs. 0.31 ± 0.07 LF/HF ratio, Recovery 3 vs. Baseline, respectively, SP: 0.86) (Fig. 2A and B). To determine the contribution of sympathetic and parasympathetic components of HRV to changes in sympathovagal balance, we evaluated each component separately (Fig. 2C). We found that the LF component of HRV was significantly reduced (LF: 17.62 ± 1.03 vs. 20.40 ± 3.49 n.u., Recovery 3 vs. Baseline, SP: 0.73) while the HF component was increased (HF: 83.95 ± 1.45 vs. 81.87 ± 1.50 n.u., Recovery 3 vs. Baseline, SP: 0.71) during the recovery period following DB + HG + OC (Fig. 2C). In addition, we evaluated the interindividual variability of LF and HF responses (Fig. 2D and E). Our data reveal that most of our subjects showed similar LF and HF spectral components during the measurement periods (Fig. 2D and E).

**Fig. 3:**
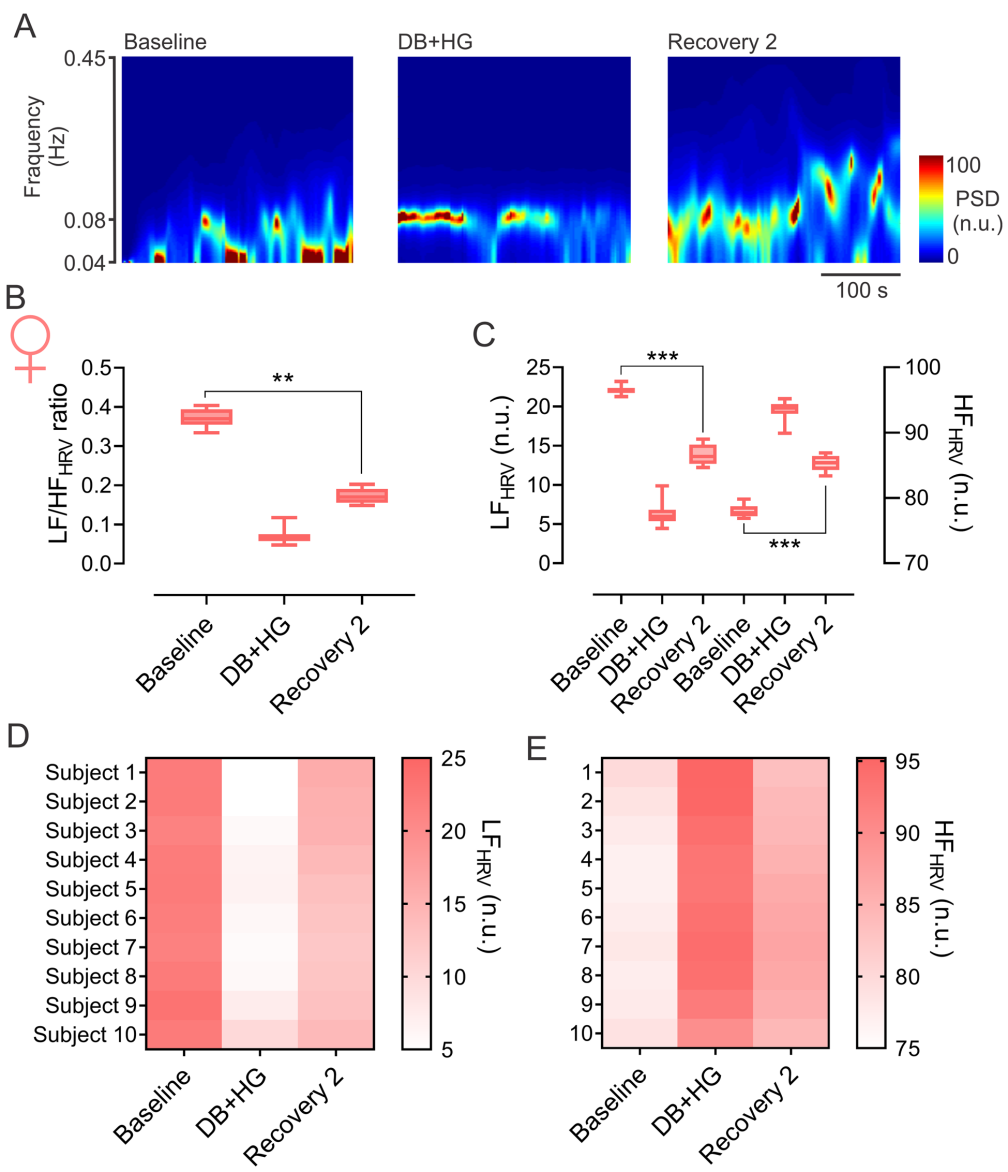
Effect of deep breathing (DB) + hand grip (DB + HG) on heart rate variability (HRV) during recovery period 2 in women. **A** Representative time-varying spectrum of HRV during baseline period, DB + HG, and recovery period 2 from one woman. Note that the power spectral density at low frequency (LF, 0.04–0.08 Hz) and high frequency (HF, 0.08–0.45 Hz) component of HRV, were increased and decreased, respectively, during recovery period 2 compared to the baseline. **B** Summary data of LF to HF ratio during baseline, DB + HG and during recovery period 2. Note that LF/HF ratio was significantly reduced in recovery period 2 compared to the baseline. **C** Summary data of LF and HF during baseline, DB + HG, and during recovery period 2. **D**, **E** Interindividual response of LF and HF of HRV in men, respectively. Data analyzed via one-way ANOVA, followed by Holm-Sidak post hoc. *p < 0.05; ***p < 0.001

Compared to men, DB + HG (recovery 2) was sufficient to elicit a significant reduction in chronotropy in women. After evaluating autonomic responses during recovery 2, we found that following DB + HG the LF/HF ratio was significantly reduced when compared to previous resting values (0.17 ± 0.02 vs. 0.37 ± 0.02 LF/HF ratio, Recovery 2 vs. Baseline, respectively, SP: 0.79) (Fig. 3A and B). Accordingly, spectral components showing the relative sympathetic and parasympathetic contribution to HRV were also significantly different during the recovery period after DB + HG (Fig. 3C). More specifically, the LF components were reduced (LF: 13.81 ± 1.26 vs. 22.09 ± 0.52 n.u., Recovery 2 vs. Baseline. SP: 0.82) while HF components were significantly increased during the recovery period following DB + HG (HF: 85.29 ± 1.19 vs. 77.93 ± 0.92 n.u., Recovery 2 vs. Baseline, SP: 0.79). Similar to what we observed in men, DB + HG had an effect on HRV spectral components in the recovery period (Fig. 3D and E).

## Discussion

In the present study we observed that: (i) women had a significant reduction in resting HR after DB + HG, which was more pronounced after DB + HG + OC, (ii) men had a reduction in resting HR only after the combination of DB + HG + OC, (iii) reductions in resting HR following intervention were accompanied by spectral shifts in HRV and, (iv) men and women both showed a predominance of vagal autonomic modulation of heart rate following DB + HG and DB + HG + OC. Considering the limited literature in the area, these results support the notion that the combination of these maneuvers may elicit beneficial reductions in resting HR and changes in cardiac sympathovagal balance, offering an readily available non-pharmacological strategy to help in cardiovascular improvements following disease-related autonomic failure.

The present study showed that the combination of two interventions was effective in reducing resting HR (study primary outcome) in a healthy population. Previous work has shown that slow breathing techniques decreases both resting HR and BP in humans with cardiovascular disease [6, 7]. Importantly, reductions in resting HR are closely related to a decreased risk of adverse cardiovascular events and all-cause mortality in patients with coronary heart disease and hypertension [7]. These salutary effects have been associated with improvements in cardiac autonomic modulation [7]. Here we reported that deep breathing strategies coupled with HG exercise with/without OC reduces resting HR and significantly shifts autonomic modulation of the heart towards parasympathetic predominance. Indeed, we observed a large increase in the parasympathetic modulation of HRV during the recovery period following DB + HG + OC in men and DB + HG in women. Thus, we speculate that in pathophysiological conditions, the combination of these strategies may offer a non-pharmacological approach to improve cardiovascular health and prognosis [6].

One of the principal mechanisms related to the beneficial cardiovascular effects of DB is activation of the baroreflex by stimulation of stretch receptors located in the aortic arch and carotid sinuses [13]. Arterial baroreceptors are activated by increases in BP which increase afferent neural discharge to central–neural–autonomic regions leading to heightened parasympathetic efferent activity through the vagus nerve to the sinoatrial (SA) node ultimately leading to decreases in HR [14]. Then, it is possible that fluctuations in intrathoracic pressure during expansion of the thorax results in fluctuations in venous filling, stroke volume, cardiac output, and peripheral blood flow (the respiratory pump) that contribute to a temporary rise in BP [15]. This in turn triggers parasympathetic activation and a subsequent decrease in HR. Importantly, studies on the effects of respiratory pattern on baroreflex function show that slow breathing at 0.1 Hz (with inspiration/expiration ratio of 1) increases baroreflex gain [16]. Unfortunately, we did not perform continuous BP recordings in our study to assess baroreflex sensitivity. Nevertheless, our data show that the HF component of HRV was markedly increased by deep breathing and isometric HG exercise. The latter has been shown to elicit brief hypertensive epochs secondary to sympathoexcitation [10, 17]. Indeed, HG exercise triggers post-exercise ischemia which in turn results in marked elevations in muscle sympathetic nerve activity, total peripheral resistance, and subsequently, BP [17]. Therefore, it is plausible that larger increases in BP may take place during concomitant deep breathing and repeated isometric muscle contractions resulting in a potentiated parasympathetic response to cope with the sympathoexcitation and pressor response elicited by DB and HG. In support of this notion, our data showed that DB and HG significantly reduced resting HR in women. However, men showed a significant reduction in resting HR only after DB and HG was coupled to OC (a more intensive stimuli for metaboreflex). The observed discrepancy between men and women may be associated with gender differences in baroreflex sensitivity [18]. Other factors may also account for gender-differences in our results like hormonal, nutritional and stress-related factors (i.e. anti-oxidant capacities). Accordingly, further studies are needed to address the contribution of baroreflex control of HR or any other factor that may contribute to the sex-differences observed in cardiac/autonomic responses following deep breathing and HG exercise.

### Limitations

Our study provide evidence showing gender-related differences in the cardiac/autonomic responses to DB, HG and OC. Although we assessed the potential of these interventions in the short-term, long-term studies are needed to gain a clearer picture of their efficacy and tolerability in humans. Another limitation is that we used indirect techniques to assess cardiac autonomic function. With that said, it is important to mention that this technique has been widely used in the clinical setting due to its low cost and non-invasive nature [10, 19]. Future studies using sympathetic nerve recordings along with pharmacological interventions are required to fully understand the cardiovascular response to DB, HG and OC. Finally, other limitation of the study is the relatively small number of subjects in which we performed the experiments. The latter should be taken into account when thinking of applying these techniques into larger cohorts of subjects. Further studies enrolling more robust number of participants will be needed in the future to validate these interventions.

## Conclusions

Our results indicate that concomitant DB + HG + OC reduces resting HR in men while DB + HG with and without OC were both effective to reduce resting HR in women. Also, DB + HG + OC in men and DB + HG in women induced marked shifts in HRV towards parasympathetic predominance. Together, these findings support the use of DB, HG and OC for cardiac autonomic modulation in healthy young adults.

## Methods

This study was approved by the ethics committee of the corporation of rehabilitation Club de Leones Cruz del Sur (Punta Arenas, Chile) (#20190719). Participants were carefully informed about the experimental procedures and the possible risks and benefits associated with their participation in the study. An appropriate signed informed consent document was obtained from all participants in accordance with the Declaration of Helsinki.

### Participants

**Fig. 4:**
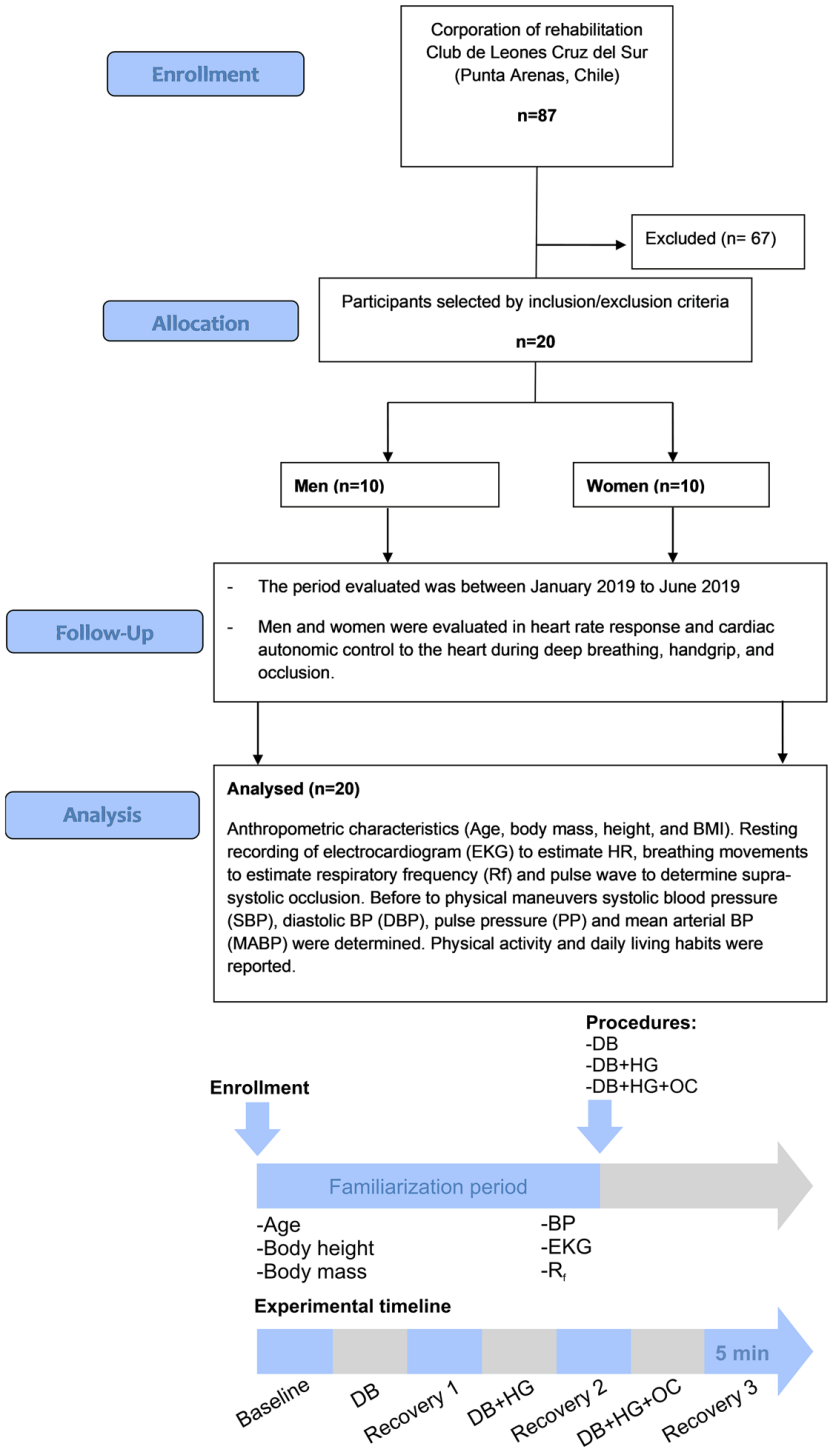
Flow chart illustrating allocation of participants and experimental description. Beginning with 87 potential participants, 67 were excluded from the study by inclusion/exclusion criteria and the study was carried out with 20 participants. From 20, 10 were men and 10 were women. After enrollment, subjects were familiarized to the procedures and body height, mass and BP were measured before the placement of ECG leads. Then, deep breathing (DB), DB + hand grip (DB + HG) and DB + HG + circulatory occlusion (DB + HG + OC) were performed in each experimental subject

Inclusion criteria for this study were as follows: (i) age between 20 and 35 years old; (ii) non-smoker; (iii) free of comorbidities including hypertension, diabetes, and heart failure; and (iv) free of arm lesions and/or arm surgeries in the 6 months prior to the study. Exclusion criteria were: (i) use of recreational drugs; (ii) taking any of the following medications: beta blockers, statins and/or any blood pressure reducing agents, anticonvulsant drug therapy, or any another drug that could influence the autonomic nervous system (i.e. Baclofen, valproic acid); (iii) pregnancy; and (iv) abnormal cardiac autonomic function at rest as determined by HRV analysis (low to high frequency ratio of HRV < 2.3) [20]. None of the participants declared to had participated in regular strength training or competitive sports activity in the 6-month period preceding the study. From 87 possible participants screened, 67 subjects were excluded, and 20 healthy individuals met the inclusion criteria and were enrolled in the study (Fig. 4). Women included in the study declared not to be under contraceptive treatment.

### Experimental procedures

All participants were familiarized with the experimental procedures one week before measurements were taken. All experimental procedures were done between 09:00 and 17:00 h in a noise-isolated room at 21–23 °C. Experimental procedures were always administered by the same qualified investigators in the same order to all participants (DB, DB + HG, DB + HG + OC). The day before experimental procedures, participants were instructed to: (i) sleep ~ 8 h and (ii) not consume coffee or other caffeine-containing beverages.

Prior to all experimental procedures, height, body mass, SBP, DBP, and 3-lead ECG were recorded. In all sessions and prior to exercises BP was determined using a sphygmomanometer (Tenso Medical Instruments Co, China) and a stethoscope (3 M, UK) by the same experienced physical therapist. MABP, PP, HR, and HRV were calculated. Height was measured using a wall-mounted stadiometer (HR-200, Tanita, Japan) and recorded to the nearest 0.1 cm. Body mass was measured to the nearest 0.1 kg using a digital scale (BF-350, Tanita, IL, USA). BMI was calculated and expressed as kg/m^2^.

Physiological experiments consisted of 5 min of seated baseline data, followed by 5 min of DB (0.1 Hz) [6, 21], followed by 5 min at rest (no paced breathing). Then, participants completed 5 min of DB + HG exercise (using their dominant hand) using a rehabilitation medical ball [10], followed by 5 min at rest. Lastly, participants completed 5 min of DB + HG (dominant hand) with a supra-systolic occlusion of their exercising dominant arm [12]. At the end of the 5 min period of DB + HG + OC, the occlusion band was released and a recovery period of 5 min was allowed. OC was attained by pumping the occlusion band to supra-systolic pressure and verified by complete disappearance of the index finger pulse wave [22].

### Deep breathing (DB)

DB exercise was performed as previously described [23]. Briefly, participants were asked to wear a nose clip on their nose to occlude both nostrils. Then, they were instructed to breathe at a rate of 0.1 Hz for 5 min with the help of a digital metronome that set each breathing cycle [21]. ECG, HR, breathing movements, and respiratory rate (R_f_) were recorded during the experiment using an analog–digital acquisition interface (PowerLab, AD Instruments, New Zealand).

### Handgrip (HG) exercise and occlusion (OC)

HG exercise and OC protocols were performed as described previously [24]. Subjects were given a rehabilitation medical ball (TH9, anti-stress ball, EYESMKT®, Chile) and were asked to perform maximal HG exercise [10]. Following the DB (only) period, subjects exercised the dominant forearm through an isometric contraction of the medical ball while they continued to perform DB for 5 min. When finished, subjects were allowed to recover for 5 min before the last training session where they performed DB + HG and OC [12, 25]. For OC, each participant was subjected to supra-systolic occlusion of the exercising arm (occlusion band located at the muscle belly of the biceps) using a rehabilitation elastic band (192 and 7.5 cm, length and width, respectively: Occlusion Cuff®, UK). OC was maintained for 5 min while the subject performed DB and HG. Pulse wave recordings from the index finger of the dominant hand in the exercising arm was performed to confirm supra-systolic occlusion as evidenced by the complete disappearance of the pulse signal [26].

### Electrocardiogram and ventilatory movements recordings

During all experimental procedures, ventilatory movements and a 3-lead ECG (35 min total time) were continuously recorded [27]. ECG electrodes were placed in DII lead according to Einthoven’s triangle with the positive electrode placed in the left leg, the negative electrode in the right hand and the ground electrode in the left arm [28, 29]. The ECG signal was low-pass (500 Hz) and high-pass (1000 Hz) filtered. The ventilatory movements were recorded with a thorax expansion device (AD Instruments, New Zealand). The inspiratory peak was used to calculate breathing rate. All recordings were digitalized and sampled at 1 kHz. HR and R_f_ were analyzed using Lab Chart Pro 8.0 software (AD Instruments, New Zealand).

### Arterial blood pressure

Prior to experimental maneuvers, SBP and DBP were determined. From SBP and DBP, MABP (1/3 of SBP + 2/3 of DBP) and PP (SBP-DBP) were calculated. Measurements were determined using a sphygmomanometer (Tenso Medical Instruments Co, China) and a stethoscope (3 M, UK) by the same experienced investigator.

### Heart rate variability (HRV)

HRV was used as an indirect measure of cardiac autonomic function [30]. From ECG recordings, RR time series were obtained, and spectral non-stationary analysis (1-min resolution) was used to obtain the power spectral density (PSD) of HRV during all experiments. Cut-off frequencies were defined as follows: very low frequency: DC-0.04; LF_HRV_: 0.04–0.08 Hz and HF_HRV_: 0.08–0.45 Hz [31]. Additionally, the LF/HF_HRV_ ratio was calculated as an indicator of sympathovagal balance. LF_HRV_ and HF_HRV_ were expressed as normalized units (n.u.). Analysis was performed within a 35 min window using Kubios HRV Premium Software v3.1 (Kubios, Finland).

### Statistical analysis

The independent variables were the different interventions (DB, HG, and OC) and the dependent variables were HR and HRV. Data is expressed as mean ± standard deviation (SD). All data were tested for normality (Shapiro–Wilk) and homoscedasticity (Levene). Data were evaluated as dictated by data structure using either an unpaired t-test or a repeated measures analysis of variance (ANOVA) (rest – DB – rest – DB + HG – rest – DB + HG + OC − recovery) followed by Holm-Sidak post-hoc analysis. Non-parametric variables were evaluated using Kruskal–Wallis analysis followed by Dunn´s post-test. The statistical power (SP, 1-β) for each significant comparison was also calculated considering arithmetic mean, standard deviation and number of subjects with an α < 0.05 (GPower, version 3.9.9.3, Dusseldorf, Germany) [32]. A p-value < 0.05 was considered statistically significant. All analyses were performed with GraphPad Prism 8.0 (La Jolla, CA).

## Data Availability

The datasets used and/or analyzed during the current study are available from the corresponding author on reasonable request.

## Abbreviations

DB: Deep breathing
HG: Hand grip
OC: Occlusion
HR: Heart rate
BP: Blood pressure
SBP: Systolic blood pressure
DBP: Diastolic blood pressure
PP: Pulse pressure
MABP: Mean arterial blood pressure
HRV: Heart rate variability
LF: Low frequency
HF: High frequency
SA: Sinoatrial
ECG: Electrocardiogram
R_f_: Respiratory frequency
PSD: Power spectral density
SD: Standard deviation
ANOVA: Analysis of variance
SP: Statistical power
BMI: Body mass index
